# Environmental Detection of SARS-CoV-2 Virus RNA in Health Facilities in Brazil and a Systematic Review on Contamination Sources

**DOI:** 10.3390/ijerph18073824

**Published:** 2021-04-06

**Authors:** Vania Aparecida Vicente, Bruno Paulo Rodrigues Lustosa, Maria Eduarda Grisolia, Caroline Pavini Beato, Eduardo Balsanelli, Viviane de Souza Gubert Fruet, Meri Bordignon Nogueira, Sonia Maria Raboni, Katherine Athayde Teixeira Carvalho, Izadora Cervelin Flôr, Morgana Ferreira Voidaleski, Ramiro Gonçalves Etchepare, Jacques F. Meis, Vanete Thomaz Soccol, Emanuel Maltempi Souza

**Affiliations:** 1 Engineering Bioprocess and Biotechnology Graduate Program, Department of Bioprocess Engineering and Biotechnology, Federal University of Paraná, Curitiba 81530-000, Brazil; brunopaulorl@ufpr.br (B.P.R.L.); duda.grisolia@gmail.com (M.E.G.); j.meis@cwz.nl (J.F.M.); vanetesoccol@gmail.com (V.T.S.); 2 Microbiology, Parasitology and Pathology Graduate Program, Department of Basic Pathology, Microbiology, Federal University of Paraná, Curitiba 81530-000, Brazil; izadoracervelinflor@gmail.com (I.C.F.); morganavoidaleski@hotmail.com (M.F.V.); 3 Laboratory of Microbiology and Molecular Biology, Department of Basic Pathology, Federal University of Paraná, Curitiba 81530-000, Brazil; caroline.pavini.beato1995@gmail.com; 4 Department of Biochemistry and Molecular Biology, Federal University of Paraná, Curitiba 81530-000, Brazil; balsanelli@gogenetic.com.br; 5 Municipal Health Secretary of Curitiba, Curitiba 80060-130, Brazil; vgubert@sms.curitiba.pr.gov.br; 6 Virology Laboratory, Clinical Hospital, Federal University of Paraná, Curitiba 80060-900, Brazil; meribordignon.nogueira@gmail.com (M.B.N.); raboni.sonia@gmail.com (S.M.R.); 7 The Pelé Pequeno Príncipe Institute, Child and Adolescent Health Research & Pequeno Príncipe Faculties and Pequeno Príncipe Hospital, Curitiba 802450-0260, Brazil; katherineteixeiradecarvalho@gmail.com; 8 Technology Sector, Department of Hydraulics and Sanitation, Federal University of Paraná, Curitiba 81530-000, Brazil; ramiro.etchepare@ufpr.br; 9 Department of Medical Microbiology and Infectious Diseases, Canisius-Wilhelmina Hospital, 6525GA Nijmegen, The Netherlands

**Keywords:** coronavirus, environmental contamination, public health, COVID-19, inanimate surfaces

## Abstract

SARS-CoV-2 environmental monitoring can track the rate of viral contamination and can be used to establish preventive measures. This study aimed to detect by RT-PCR the presence of SARS-CoV-2 from inert surface samples in public health settings with a literature review about surface contamination and its burden on spread virus. Samples were collected from health settings in Curitiba, Brazil, between July and December 2020. A literature review was conducted using PRISMA. A total of 711 environmental surface samples were collected from outpatient areas, dental units, doctors’ offices, COVID-19 evaluation areas, and hospital units, of which 35 (4.9%) were positive for SARS-CoV-2 RNA. The frequency of environmental contamination was higher in primary care units than in hospital settings. The virus was detected on doctors’ personal items. Remarkably, the previously disinfected dental chair samples tested positive. These findings agree with those of other studies in which SARS-CoV-2 was found on inanimate surfaces. Detection of SARS-CoV-2 RNA on surfaces in public health settings, including those not meant to treat COVID-19, indicates widespread environmental contamination. Therefore, the intensification of disinfection measures for external hospital areas may be important for controlling community COVID-19 dissemination.

## 1. Introduction

On 30 January 2020, the World Health Organization (WHO) drew the world’s attention to an outbreak of a new coronavirus disease (COVID-19) caused by severe acute respiratory syndrome coronavirus 2 (SARS-CoV-2). Initially identified in Wuhan, China (Hubei Province), within 3 months COVID-19 occurred worldwide accounting for thousands of deaths, thus leading to WHO to declare it a global pandemic on 11 March 2020 [1]. On 3 February 2020, the Brazilian Ministry of Health declared a national public health emergency. By the end of 2020, Brazil was among the three countries with the highest number of cases and fatalities worldwide [2], underscoring an extreme health emergency.

Human-to-human transmission has been reported, with an incubation period of 2 to 10 days. The virus spreads through contaminated droplets, contamination of hands by direct contact, or indirectly via inanimate surfaces [3,4]. The most significant concern regarding SARS-CoV-2 is its transmission through aerosols and direct contact [5], which is associated with environmental conditions and human behavior [6,7]. However, several studies reported a correlation between the number of daily confirmed cases of COVID-19 and the environmental viability of the virus [8,9]. Therefore, the spread control of SARS-CoV-2 is a global challenge, and the evidence of its circulation on the inanimate surfaces are important to reinforce public health measures to limit transmission of the virus [5,10,11,12,13].

According to the WHO, there is not enough information regarding the persistence of SARS-CoV-2 on surfaces [7]. The virus appears to behave like other coronaviruses, which can survive on inanimate surfaces for at least several hours [6,14] and under various conditions of temperature, humidity, and pH [8]. Studies based on the detection of viral RNA have reported the persistence on metal, glass, and plastic, at room temperature for hours or even days [8,14]. The stability of SARS-CoV-2 has been reported to be higher on smooth surfaces [15]. Furthermore, Ye et al. [16] detected the environmental presence of virus RNA on objects in medical centers. The study showed contamination in various patient care areas and emphasized the need for adequate environmental cleaning.

Recently, Lewis [17] presented an extensive discussion concerning environmental surfaces as a potential infection source. The author remarks that evidence of transmission from indirect contact with contaminated inanimate surfaces is limited compared to other routes of infection, such as through droplets and aerosols. However, emerging data suggest that the SARS-CoV-2 virus can spread and persist in the environment and be transferred from inert surfaces to human hands, leading to autoinoculation of the mucous membranes of the nose, eyes, or mouth [8,14]. Moreover, the nosocomial transmission of SARS-CoV-2 has been reported [18,19].

According to Santarpia et al. [20], effective measures for the control of emerging infectious diseases require a solid understanding of modes of transmission. In addition, the WHO recommended that environmental surveillance research should be considered as an important public health objective to advance knowledge about COVID-19. Thus, environmental RNA detection can be a strategy of public health institutions to track and monitor the rate of viral spread in communities and to suggest preventive measures [14,21,22].

In this context, this study aimed to investigate the presence of SARS-CoV-2 RNA on inert surfaces in different settings of the public health system in Curitiba, Brazil to evaluate possible sources of environmental contamination. In addition, a systematic review of the literature was undertaken to compare our results and provide a critical discussion about the environmental risks of infection.

## 2. Materials and Methods

### 2.1. Molecular Analysis

#### 2.1.1. Sampling Areas 

**Figure 1:**
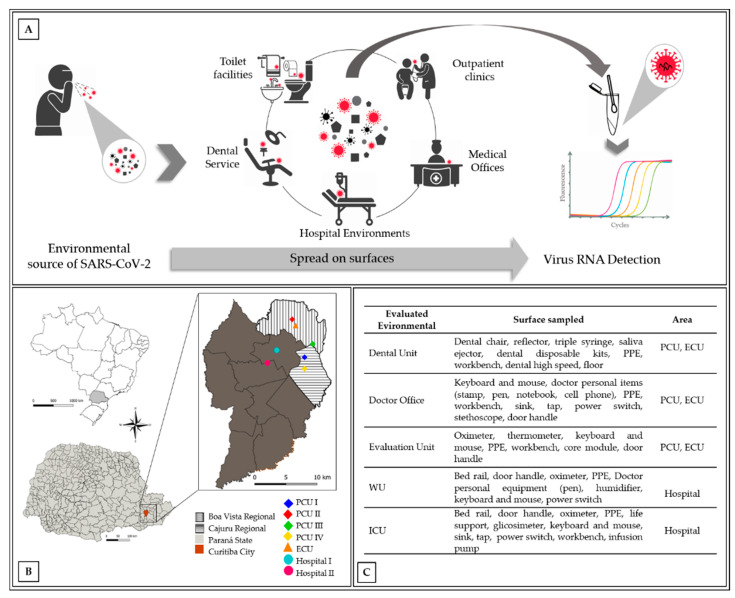
Distribution of sampling sites. (**A**) Sampling conducted according to surfaces more frequently touched in the health care facilities; (**B**) location map of the sampling; (**C**) description of environment and surfaces sampled. Notes: PCU = primary care unit; ECU = emergency care unit; WU = hospital ward unit; ICU = hospital intensive care unit; PPE = personal protective equipment. I, II, III, and IV represent the number of primary care units evaluated.

The investigation was performed in Curitiba, State of Paraná, southern Brazil. The sampled locations are shown in Figure 1b. Recommendations on the window of infectivity and detection potential were based on studies by Wu et al. [23] and Ahmed et al. [6,24]. The first confirmed case of COVID-19 in Curitiba was on 11 March 2020, with an average increase from 100 to 120 new cases/week between April and May. A considerable increase to 1017 cases per week was observed in June and further increased to an average of 3,537 and 3514 new cases/week in July and August, respectively. However, in November, a second wave of new cases resulted in 100,482 notifications by the end of 2020.

The study samples were collected during the two high waves of COVID-19 infection from July 2020 to December 2020. Samples were obtained from four primary care units (PCUs; I, II, III, and IV), one emergency care unit (ECU), and two public hospitals, including COVID-19 ward units (WUs) and intensive care units (ICUs). The PCUs receive patients for minimal medical intervention, whereas the ECU receives patients who require mild-to-moderate intervention. Both groups received patients with and without COVID-19. The dental units and doctors’ offices at the ECU and PCU were also evaluated (Figure 1A,C).

Sterile rayon swabs were performed on the entire surface sampled and stored in a 5% sodium dodecyl sulfate (SDS) solution [16]. Large surface areas were partially sampled, considering areas of frequent exposure. For instance, in the dentist’s chair, we evaluated the headrest, feet contour, arms, and reflectors. On the computer keyboard, all keys were swabbed. Likewise, in the chairs of the offices, the arms were selected as collection points. Finally, on the X-ray bucky wall, the chin support location and the region of the head backrest surface were assigned. The samples were collected in patient rooms, bathrooms, waiting rooms, general wards, and ICUs before and after routine cleanings. 

Environmental cleaning of surfaces was undertaken by applying water and detergents with commonly used hospital-level disinfectants such as sodium hypochlorite (0.1–0.5%), ethanol (62–71%), and the disinfectant based on alkyl dimethyl benzyl ammonium chloride (benzalkonium chloride) 5.2%, and polyhexamethylene biguanide (PHMB) 3.5%. The cleaning products and disinfectants are used according to specific areas and material in one or two steps according to Brazilian Health Regulatory Agency (ANVISA) protocols [25]. The last two are the most used for disinfecting surfaces in health care units and hospital settings, such as in ICU and ward units. PHMB benzalkonium chloride has been intensively used in COVID-19 care units.

During the first wave, samples were collected from the PCU and the ECU. In addition, samples from hospitals were included during the second wave, and multiple sampling was conducted on the surfaces with the highest positivity rate in the first wave.

#### 2.1.2. Detection of Viral RNA on Inert Surfaces

The samples were transported on an ice pack in a 5% SDS solution to the CMRP/Taxonline (https://www.cmrp-taxonline.com) at the Federal University of Parana (UFPR). RNA was immediately extracted using the MagMAX™ Viral RNA Isolation Kit (Thermo Fisher, Carlsbad, CA, USA) according to the manufacturer’s instructions. RNA purity was evaluated by spectrophotometry (NanoDrop^®^, Thermo Scientific, Waltham, MA, USA).

The Polymerase Chain Reaction in Real Time (RT-PCR) kit used for the samples was the BIOMOL OneStep/COVID-19 kit [26]. It uses two SARS-CoV-2 virus targets: the conserved Orf1ab region and the *N* gene. The amplification of the pathogen’s genetic material, combined with the amplification of internal control, indicating viral RNA in the sample. The internal control kit contained a negative control (NTC—Water) and a positive control (plasmid—derived from class I GMOs) to confirm the results. The virus detection reactions were performed in blocks of 94 samples plus the control. Control assays were performed to determine the minimum necessary RNA of the environmental samples for successful detection by RT-PCR. The test showed that 2 ng/µL was the concentration of RNA required for accurate detection of SARS-CoV-2.

### 2.2. Statistical Analysis

Data were analyzed using the statistical software R (version 4.0.2). The data were previously submitted to a binomial factor using a logistic regression model and a multivariate analysis (ANOVA) to find significant differences between the frequency of positive RT-PCR to SARS-CoV-2 and influencing factors, such as data sampling, location, healthcare unit characteristics, contact with COVID-19 patients, and the disinfection protocol used. In this manner, a pairwise analysis was performed using Tukey’s multiple comparison test with a confidence interval of 95%.

### 2.3. Systematic Review: Search Strategy and Selection Criteria 

The systematic review followed the guidelines established by the Preferred Reporting Items for Systematic Reviews and Meta-Analyses (PRISMA) [27]. The bibliographic research included four databases (Medline, Scopus, LILACS, and SciELO) using the question: what is the most common surface contaminated with SARS-CoV-2 in healthcare facilities? With following terms in Medline: ‘Environment’ (mesh) or ‘Gene-Environment Interaction’ (mesh), and ‘COVID-19’ (supplementary concept) and ‘severe acute respiratory syndrome coronavirus 2’ (Supplementary Concept); Scielo: environment or gene-environment interaction and COVID-19 and severe acute respiratory syndrome coronavirus 2; LILACS: environment or gene-environment interaction and COVID-19 and severe acute respiratory syndrome coronavirus 2; Scopus: environment or gene-environment and interaction and COVID-19 and severe and acute and respiratory and syndrome and coronavirus 2.

The inclusion criteria were (a) studies related to environmental sampling in health care facilities using sterile swabs, (b) followed by RNA extraction, and (c) RT-PCR for environmental surface sampling. Editorials, reviews, commentaries, brief communications, opinion pieces, and papers that did not meet the inclusion criteria were excluded.

The final search was conducted on 16 October 2020. The results from the databases were merged and duplicates were removed. Two authors independently assessed the combined results of the electronic database search, and discrepancies were discussed and agreed upon according to the inclusion and exclusion criteria. Additional articles of interest were identified by reviewing the bibliographies of relevant articles. 

The literature review resulted in 1527 bibliographic references with 928 from Medline, 624 from Scopus, four from Lilacs, and no data from SciELO. In addition, 11 data points were gathered from other sources. After removing duplicates, 1505 results remained for screening, of which 46 eligible references were assessed for the final analysis (Supplementary Figure S1).

The meta-analysis was conducted using the ‘metafor’ package to test the heterogenicity and the odds ratio of the data [28]. A heterogenicity test was conducted to analyze the variability of the literature data in relation to viral RNA detected on environmental surfaces and viral RNA particles present in aerosol air samples. *I*^2^ statistics and Cochran’s Q test were used to assess statistical heterogeneity with a 95% confidence interval.

## 3. Results

A total of 711 environmental surface samples were collected, which included 234 from dental units, 177 from doctor’s offices, 160 from COVID-19 evaluation units, 45 from COVID-19 hospital WUs, 55 from ICU, and 40 from bathrooms from outpatient health units (Figure 1A,C). In the dental units, viral RNA was found on the dental saliva ejector, dental triple syringe, and disposable dental kits. The RNA virus was also detected on non-disposable instruments, such as dental reflectors and dental chairs. In addition, the dental armchair was found to be positive for SARS-CoV-2 RNA even after disinfection (Table 1).

In the doctor’s offices, viral RNA was detected on personal items such as pens, stamps, and notebooks. Moreover, door handles, computer keyboards, mice, armchairs, and oximeters were positive for SARS-CoV-2. In the COVID-19 evaluation unit, it was found on keyboards and mice, oximeter, thermometer, patient armchair, door handle, and the X-ray bucky wall (Supplementary Table S1). 

Of the 711 samples analyzed, 35 samples were positive independent of the gene detected by RT-PCR. Within that, 21 were positive only for the *Orf1* gene, with threshold cycle values (Ct value) ranging from 22.74 (from the dental chair and dental triple syringe) to 39.43, and 19 were positive only for the *N* gene, with Ct values ranging from 31.88 (from the PCU’s sink) to 39.92 (Table 1).

**Figure 2:**
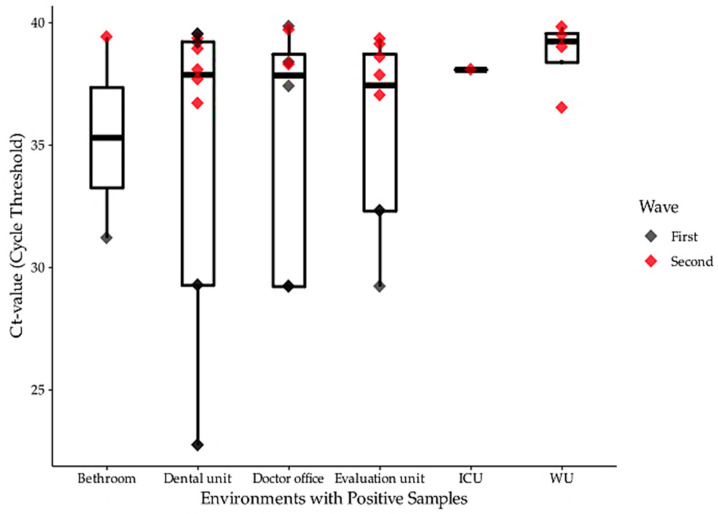
Ct value range detected during the first and the second wave of the COVID-19 pandemic in Curitiba, south of Brazil according to the health service. Notes: health service setting according to the public health system from Curitiba: ICU = intensive care unit; WU = ward unit. Black and red diamonds represent the positive sampling points during the first and second waves, respectively.

Moreover, among the 711 samples analyzed, the Ct values detected by RT-PCR were higher in the samples collected during the second wave (Figure 2). The frequency of environmental contamination was higher in the PCUs than in the other units, with a frequency of 4.11% of *Orf1* contamination, compared to 2.7% and 1.28% in the hospital environment and the ECU, respectively (F(2) = 2.058, *p* = 0.092, Table 2). Regarding the detection of the *Orf1* gene, there was an association between positive RT-PCR and the location of sampling, with the PCU-II more likely to have a positive result than the others (F(4) = 5.264, *p* < 0.001, Table 2). A significant correlation was found between *N* gene detection and presence of COVID-19 patients (F(2) = 9.151, *p* < 0.001, Table 2).

Our systematic literature review selected a total of 22 papers that included detection of SARS-CoV2 in environmental samples; most cases (95.5%) were from surfaces in hospital COVID-19 units. The majority of the studies were conducted in China (50%) and Italy (18.2%) with two studies from Singapore, South Korea and one study from Iran, the United States, and Brazil. Many of the collected samples from COVID-19 units in hospitals were taken in proximity to SARS-CoV-2 RT-PCR positive patients (Supplementary Table S2). Considering these data, it was possible to identify certain surfaces in hospital units where SARS-CoV-2 contamination was more prevalent, such as bed rails, door handles, and medical equipment (Supplementary Figure S2).

**Figure 3:**
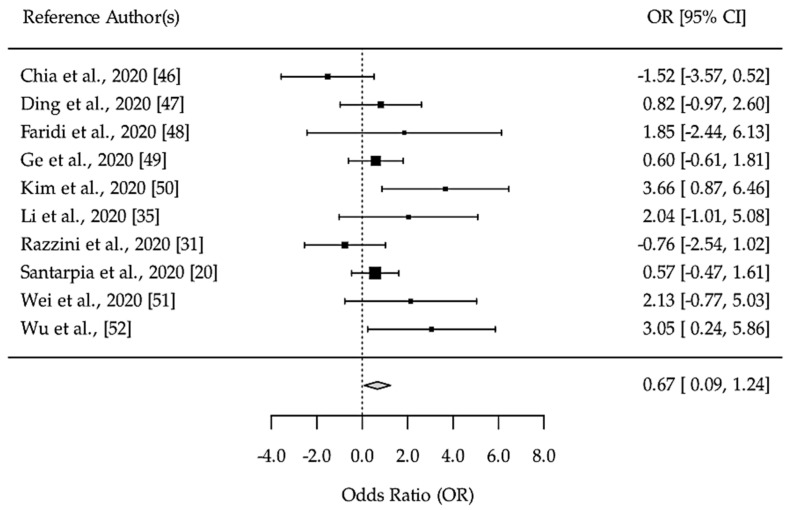
Fixed-effects meta-analysis of studies in the comparison between environment surface sampled and aerosols sampled positive RT-PCR. Notes: Odds ratio is derived from positive and negative RT-PCR by SARS-CoV-2 in surface and aerosol samples reported by each study. OR = odds ratio test; [95%CI]—confidence interval of 95%. Reference author(s)—first author surname of each study included in the metanalysis; The square’s sizes represent the weight of each study included in the analysis; the gray diamond represents the mean odds ratio of the comparison.

From the data gathered in the literature review, our meta-analysis used 10 studies to compare the odds of surface contamination in relation to aerosol contamination. According to the fixed effect model (Figure 3), viral RNA had a higher probability of being positive in the RT-PCR on the environmental surfaces than in the aerosol samples (OR = 0.67, CI_95_ = 0.09–1.24, *p* = 0.023). Heterogeneity in effect size between the studies was low (I^2^ = 44.36%, H2 = 1.80, Q_(9)_ = 16.177, *p* = 0.063). In addition, a significant correlation was not observed between gene target used to detect SARS-CoV-2 on environmental surfaces and the frequency of positive RT-PCR results (*p* = 0.3529, Supplementary Table S2).

## 4. Discussion

The molecular analysis of viral RNA from environmental surfaces in public health settings in our study showed that the virus can be detected on routine equipment and on physicians’ personal items which unknowingly become carriers of SARS-CoV-2. Furthermore, it was also detected in the dental offices, with the virus present on the reflector, chair, and saliva ejector. Remarkably, the virus was persistent in some dental office samples even after disinfection procedures were employed, such as those for the triple syringe, reflector, and chair. This is the first study to focus on public health including dental services. These results showed that the risk of cross-infection within the dental office should be a concern. Likewise, the literature points out that the risk may be high between dentists and patients because of the peculiarity of dental practice, including the surface contamination hotspots, where virus-laden droplets tend to deposit [29,30].

According to the data presented in Figure 2, the rates of the viral load/samples obtained months later (as observed during the second evaluation) reduced to 2.3% (*n* = 8/348) after the intensifying cleaning and disinfection procedures with an intermediate level disinfectant based on benzalkonium chloride and PHMB. Although the accumulation of the virus in hotspots may be troublesome, coronaviruses can be inactivated easily using common disinfectants, such as ethanol, sodium hypochlorite, and hydrogen peroxide [14]. For instance, for the disinfectant used based on benzalkonium chloride (PHMB), it was observed that a higher cleaning frequency at reduced intervals adopted, can explain our findings.

Previous studies have reported that SARS-CoV, middle east respiratory syndrome (MERS-CoV), and influenza viruses can survive on inert surfaces for long periods [31,32], although the use of molecular detection methods might not necessarily represent the presence of viable virus [31]. As the viability of SARS-CoV-2 on inert surfaces has not been well studied yet, more understanding of transmission, viral load dynamics, duration of human viral shedding, and environmental persistence should be thoroughly investigated. 

When considering the hospital environment, our study demonstrated that bed rails and door handles are the most commonly contaminated surfaces in the WUs comprising 22% and 14% of the total positive contaminated samples, respectively. In the ICU only the bed rail was positive which was also demonstrated by Ye et al. [16], who reported that frequently touched surfaces could spread the virus. Likewise, Razzini et al. [31] reported interesting data on tracking of the SARS-CoV-2 virus in environmental hospital samples with a positivity rate of 24.3% and no positive results in clean areas. These data indicate the importance of rigorous disinfection and protective measures. 

Furthermore, Ryu et al. [33] showed that person-to-person transmission was an essential route for the COVID-19 outbreak, which can be intensified if health professionals are infected. These authors consider close contact with surfaces contaminated with SARS-CoV-2 to be one of the possible routes of transmission, in addition to person-to-person contact. In this context, our study showed a high incidence of positive samples in the X-ray room, especially on equipment directly in contact with the patient, suggesting the equipment is an infection risk.

The systematic review showed that bed rails, door handles, and floor were the most common contaminated surfaces in proximity to the COVID-19 units (Supplementary Figure S2). Hu et al. [34] collected samples from COVID-19 wards and showed that as sampling of the floor moved away from the patient’s bed, the frequency of positive samples decreased. The sampling positivity on environmental surfaces ranged from 2.2% [35,36] to 74.2% [20] according to the surface evaluated and the sampling location [3,37]. In our environmental inanimate surface evaluation, a positivity rate of 4.9% was observed using random sampling with some positive samples in hospital wards in up to 8%. In a recent study in the central region of Brazil [38], the authors reported 5.25% positive samples, although they used only the *N* gene as a target to evaluate diverse environments. Together, these results indicate a high prevalence of infected patients in the sampled settings and emphasize the need for thorough decontamination of areas with frequent transit of potentially infected patients. 

The statistical analysis based on our systematic literature review did not show significant differences in relation to the target gene used. Most reported environmental studies on the proximity of hospitalized COVID-19 patients used different genes target such as *E*, *RdRp*, *Orf1b*, and *N*. Nevertheless, our environmental investigation showed a significant increase in positive environmental samples with Ct values from 22.74 to 39.92 when the results from both *Orf1b* and *N* target genes were combined (Figure 2). Thus, our results indicate that the environmental detection of SARS-CoV-2 must be carried out focusing on at least two genes.

Eslami and Jalilli [15] discussed the effects and roles of environmental factors (climate change, water, air, and food transfer) and disinfection of surfaces and hands in the transmission and prevalence of viruses in the environment. The literature emphasizes environmental dynamics and persistent viral infectivity [8,35,39,40,41,42]. According to Marquès and Domingo 2021 [43] despite most reported data on SARS-CoV-2, inanimate surfaces are revisions of the scarce data and/or approaches based on data from other human coronaviruses, recently, several studies on the stability and infectivity of SARS-CoV-2 showed evidence of surface stability of SARS-CoV-2.

However, few studies have evaluated the presence of viable viruses from RT-PCR positive environmental samples [20,37,44,45]. Santarpia et al. [20] observed viral replication in cell cultures of samples collected from rooms of patients infected with SARS-CoV-2 confirming the potential infectiousness of the virus detected in the environment with a Ct value of <36.5. Although the influence of viral load on transmissibility via environmental samples has been extensively discussed there is no data showing viable virus in samples with Ct value > 36.5 [3,20]. Nonetheless, it is essential to note that various technical factors can affect virus viability and, consequently, its isolation in cell cultures. Therefore, a negative cell culture may not mean the absence of infectious viruses, suggesting further data are required to elucidate this. 

Regarding the presence of viral RNA on surfaces in outpatient and hospital units, our findings demonstrated that the positivity of RT-PCR in samples collected in outpatient clinics was higher than that in hospital environments. This data emphasizes the need to implement more stringent disinfection measures in these areas, where there was the circulation of a greater number of people and the use of personal protective equipment was less frequent. This highlights the importance of monitoring environmental contamination as a means of reducing transmission and providing an early warning of areas contaminated by SARS-CoV-2. 

Data from our systematic review suggest that easily touched surfaces are more often positive for viral RNA than aerosol samples [20,31,35,46,47,48,49,50,51,52]. In addition, the meta-analysis supports that surface samples are 67% more frequently contaminated with viral RNA than aerosol samples (Figure 3). However, the data already reported [10] have been demonstrated that the virus is primarily spread through contact and respiratory droplets and so, more studies are needed to assess the significance of indirect transmission of SARS-CoV-2.

## 5. Conclusions

In conclusion, these data are relevant to the understanding of environmental contamination around COVID-19 as it is among the few comprehensive and long-term sampling studies available to public health settings. RNA detection revealed that some surfaces can be considered at increased risk of infection once the viral RNA was detected even after disinfection procedures. Moreover, these results indicate widespread environmental contamination and demonstrated the relevance of environmental viral RNA tracking to identify the focus of infection, including in health units not meant to treat COVID-19. Therefore, the intensification of disinfection measures for external hospital areas may be important for the surveillance and control community COVID-19 dissemination.

## Figures and Tables

**Table 1:** RT-PCR positive samples obtained from July 2020 to December 2020.

Location	Month	Sample Description	Pd	CT—*Orf1*	CT—*N*
PCU	July	Dental triple syringe	No	-	39.18
PCU	July	Sink and tap from the evaluation unit	No	30.51	31.88
PCU	July	Dental chair	Yes	22.74	-
PCU	July	Dental triple syringe	Yes	22.74	-
PCU	August	Oximeter from the doctor room	No	38.38	-
PCU	August	Patient armchair	No	-	39.84
ECU	August	Oximeter from the evaluation unit	No	32.31	-
ECU	August	Thermometer from the evaluation unit	No	32.31	-
ECU	September	Dental reflector	No	-	39.53
ECU	September	Dental saliva ejector	No	-	39.53
PCU	September	Dental disposable kit	No	29.27	-
PCU	September	Keyboard and mouse from the doctor room	No	29.22	-
PCU	September	Doctor personal item—notebook	No	29.22	-
PCU	September	Workbench disposable gloves workbench	No	29.27	-
PCU	September	Doctor personal item—pen and stamp	No	29.22	-
PCU	September	Patient armchair from the doctor room	No	37.40	-
PCU	September	Keyboard and mouse from the evaluation unit	No	29.22	-
ECU	December	Doctor personal item—stamp	No	-	39.70
ECU	December	Door handle from inside of the doctor room	No	-	38.29
ECU	December	Toilet discharge from the evaluation unit	No	-	39.41
PCU	December	Patient armchair from the covid-19 evaluation unit	No	-	39.12
PCU	December	Keyboard and mouse from the covid-19 evaluation unit	No	37.03	-
PCU	December	Doctor personal item—pen from the covid-19 evaluation unit	No	-	38.59
PCU	December	Dental chair	Yes	36.87	38.48
PCU	December	Dental reflector	Yes	-	39.35
PCU	December	Dental saliva ejector	No	-	38.93
PCU	December	Dental saliva ejector	No	38.07	-
PCU	December	Dental disposable kit	No	36.70	-
WU	December	Bed rail from the hospital covid-19 ward	No	38.08	39.92
WU	December	Sink and Tap of the hospital covid-19 ward	No	36.13	36.91
WU	December	Door handle from the hospital covid-19 ward	No	-	39.48
ICU	December	Bed rail from the hospital intense care unit	No	38.08	-
WU	December	Life support—humidifier from the hospital covid-19 ward	No	-	39.82
ECU	December	X-ray bucky wall	No	-	37.85
ECU	December	Door handle from the X-ray room	No	39.43	39.25

Notes: PCU = primary care unit; ECU = emergency care unit; WU = COVID-19 ward unit: hospital; ICU = COVID-19 intensive care unit; Pd = previous cleaning with alcohol 70°, or disinfectant provided by the public health service; in this case, we ensured that the sample was made after (Yes) or before (No) any disinfection.

**Table 2:** Comparison between the positive samples collected from the environmental surfaces of the health facilities, the service unit, the site of sampling, the month of sampling, presence/absence of patients with symptoms of COVID-19, and with sampling before and after cleaning procedures.

Sample N°		*N* Gene	%	IC (95%)	*p*	*Orf1* Gene	%	IC (95%)	*p*
*Health Unit*									
PCU	365	8	2.19	2.18–2.19	0.668	16	4.38	4.38–4.39	0.092
ECU	235	7	2.98	2.98–2.98		3	1.28	1.27–1.28	
Hospital	111	4	3.60	3.60–3.61		3	2.70	2.70–2.71	
*Health place*									
PCU I	78	4	5.13	5.15–5.11	0.063	2	2.56	2.53–2.60	<0.001
PCU II	57	0	0.00	−0.02–0.02		8	14.04	14.00–14.07	
PCU III	22	1	4.55	4.53–4.56		2	9.09	9.06–9.13	
PCU IV	208	3	1.44	1.42–1.46		4	1.92	1.89–1.96	
ECU	235	7	2.98	2.96–3.00		3	1.28	1.24–1.31	
Hospital 1	73	1	1.37	1.35–1.39		1	1.37	1.33–1.41	
Hospital II	38	3	7.89	7.88–7.91		2	5.26	5.23–5.30	
*Evaluated Environment*									
Dental Unit	234	6	2.56	2.54–2.58	0.051	7	2.99	2.99–3.00	0.763
Doctor office	177	3	1.69	1.67–1.71		6	3.39	3.38–3.40	
Evaluation Unit	160	4	2.50	2.48–2.52		5	3.13	3.12–3.13	
WU	45	4	8.89	8.87–8.91		2	4.44	4.44–4.45	
ICU	55	0	0.00	−0.02–0.02		1	1.82	1.81–1.82	
Bathroom	40	2	5.00	4.98–5.02		1	2.50	2.49–2.51	
*Month of sampled*									
July	83	2	2.41	2.40–2.42	0.412	3	3.61	3.60–3.63	0.525
August	176	1	0.57	0.56–0.58		3	1.70	1.69–1.72	
September	104	2	1.92	1.92–1.93		8	7.69	7.68–7.71	
December	348	14	4.02	4.02–4.03		8	2.30	2.29–2.31	
*Presence of COVID-19 patient*									
Positive	411	10	2.43	2.42–2.44	<0.001	14	3.41	3.40–3.42	0.938
Negative	24	0	0.00	−0.01–0.01		0	0.00	−0.01–0.01	
Indifferent	276	9	3.26	3.25–3.27		8	2.90	2.89–2.91	
*Disinfected surface*									
Yes	206	2	0.97	0.96–0.98	0.224	4	1.94	1.93–1.95	0.149
No	505	17	3.37	3.36–3.38		18	3.56	3.56–3.57	

Notes: Service unit: PCU = primary care unit, ECU = emergency care unit; WU = COVID-19 ward unit; ICU = COVID-19 intensive care unit; Sample N° = number of samples from each category; Gene *N* = number of positive RT-PCR from each category for the *N* gene; % = frequency of positive RT-PCR considering the Sample N°; *Orf1* Gene = Number of positive RT-PCR from each category for the *Orf1* gene; IC (95%) = confidence interval as 95%, considering a normal distribution of the sample; *p* = significant values for *p* < 0.05.

## Data Availability

The data presented in this study are available upon request from the corresponding author.

## Supplementary Materials

The following are available online at https://www.mdpi.com/article/10.3390/ijerph18073824/s1, Supplementary Table S1. Prevalence of Sars-CoV-2 on surfaces sampled in primary care units, emergence care units, and hospitals from Curitiba, South of Brazil; Supplementary Table S2. Bibliographic review: Studies that evaluated environmental surfaces for contamination with SARS-COV-2; Supplementary Figure S1. Flowchart of search methods for bibliographic review; Supplementary Figure S2. Frequency of citation of the contaminated surface in the published data; Supplementary Table S3. PRISMA 2009 Checklist.
