# Supplementary material for: Environmental Detection of SARS-CoV-2 Virus RNA in Health Facilities in Brazil and a Systematic Review on Contamination Sources
Source: Int J Environ Res Public Health. 2021 Apr 6;18(7):3824. doi: 10.3390/ijerph18073824 (PMC8038740; doi:10.3390/ijerph18073824)
Supplement: Supplementary file 1 [file ijerph-18-03824-s001.pdf]

Supplementary material

Supplementary Table S1 – Prevalence of Sars-CoV-2 on surfaces sampled in Primary care units, Emergence care units and Hospital from Curitiba, South of Brazil

Surface sampled	Gene *N*	%	IC (95%)	Gene *Orf1*	%	IC (95%)
Workbench	0/19	0·0	-0·14 - 0·14	1/19	5·3	5·19 - 5·34
Chair	2/68	2·9	2·80 - 3·09	4/68	5·9	5·81 - 5·96
Doctor personal items	2/60	3·3	3·19 - 3·48	1/60	1·7	1·59 - 1·74
Keyboard and Mouse	0/88	0·0	-0·14 - 0·14	3/88	3·4	3·34 - 3·48
PPE	0/74	0·0	-0·14 - 0·14	0/74	0·0	-0·07 - 0·07
Closet and refrigerator handle	0/5	0·0	-0·14 - 0·14	0/5	0·0	-0·07 - 0·07
Health care core module	0/15	0·0	-0·14 - 0·14	0/15	0·0	-0·07 - 0·07
Dental chair	1/46	2·2	2·03 - 2·32	1/46	2·2	2·10 - 2·25
Dental high speed	0/17	0·0	-0·14 - 0·14	0/17	0·0	-0·07 - 0·07
Dental disposable kit	0/16	0·0	-0·14 - 0·14	2/16	12·5	12·43 - 12·57
Dental reflector	2/22	9·1	8·95 - 9·24	0/22	0·0	-0·07 - 0·07
Dental triple syringe	1/21	4·8	4·62 - 4·91	1/21	4·8	9·45 - 9·60
Dental saliva ejector	2/23	8·7	8·55 - 8·84	1/23	4·3	4·27 - 4·42
Door handle	3/21	14·3	14·14 - 14·43	1/21	4·8	4·69 - 4·84
Power switch	0/7	0·0	-0·14 - 0·14	0/7	0·0	-0·07 - 0·07
Toilet bowl	1/18	5·6	5·41 - 5·70	0/18	0·0	-0·07 - 0·07
Sink and Tap	2/66	3·0	2·89 - 3·18	2/66	3·0	1·44 - 1·59
Oximeter	0/26	0·0	-0·14 - 0·14	2/26	7·7	7·62 - 7·77
Stethoscope	0/25	0·0	-0·14 - 0·14	0/25	0·0	-0·07 - 0·07
Thermometer	0/14	0·0	-0·14 - 0·14	1/14	7·1	7·07 - 7·22
Life support	1/8	12·5	12·34 - 12·64	0/8	0·0	-0·07 - 0·07
Pressure gauge	0/8	0·0	-0·14 - 0·14	0/8	0·0	-0·07 - 0·07
Infusion pump	0/3	0·0	-0·14 - 0·14	0/3	0·0	-0·07 - 0·07
Glycosometer	0/1	0·0	-0·14 - 0·14	0/1	0·0	-0·07 - 0·07
Bed rail	1/9	11·1	10·97 - 11·26	2/9	22·2	22·15 - 22·30
X-Ray room	1/2	50·0	49·86 - 50·14	0/2	0·0	-0·07 - 0·07
Hospital Purge	0/2	0·0	-0·14 - 0·14	0/2	0·0	-0·07 - 0·07
Examination ward floor	0/14	0·0	-0·14 - 0·14	0/14	0·0	-0·07 - 0·07
General Surfaces	0/13	0·0	-0·14 - 0·14	0/13	0·0	-0·07 - 0·07
Total	19/711	2·7		22/711	3·1	

Notes: Surface sampled – description of the surface sampled; Gene N – Ratio between number of each environment surface sampled and number of positive RT-PCR by the gene N; Gene Orf1 – Ratio between number of each environment surface sampled and number of positive RT-PCR by the gene *Orf1*; IC (95%) - confidence interval as 95%, considering a normal distribution of the sample;

Supplementary Table S2 - Bibliographic review: Studies that evaluated environmental surfaces for contamination with SARS-COV-2.

Author	Country	Sampling site	Sample Nº	%	Target genes[A]	Low-CT
Abrahão et al., 2020 [36]	Brazil	A, J	933	5.5	*N1, N2*	23.3
Chia et al., 2020 [39]	Singapore	A	245	26.5	*Orf1ab, E*	20.37
Colaneri et al., 2020 [35]	Italy	A	26	7.6	*E*	-
Ding et al., 2020 [40]	China	A, H	107	6.5	-	36.20
Faridi et al., 2020 [41]	Iran	A	10	10.0	*E, RdRp*	-
Ge et al., 2020 [42]	China	A, B, C	112	16.9	*N*	25.62
Hu et al., 2020 [32]	China	A	46	28.2	*Orf1ab*	26.00
Huang et al., 2020 [14]	China	A	96	3.1	*Orf1ab, N*	35.00
Kim et al., 2020 [43]	South Korea	A	330	26.9	*E, RdRp*	25.00
Li et al., 2020 [33]	China	A	90	2.2	-	-
Lv et al., 2020 [4]	China	C	61	0.0	*Orf1ab, N*	-
Pasquarella et al., 2020 [23]	Italy	A	15	26.6	*E*	31.00
Razzini et al., 2020 [29]	Italy	A	37	24.3	-	21.50
Rimoldi et al., 2020 [37]	Italy	D, E	18	44.4	*Orf1ab, N, E*	-
Ryu et al., 2020 [31]	South Korea	A	79	16.4	*N, E, RdRp*	30.28
Santarpia et al., 2020 [21]	USA	A	163	72.4	*N, E*	15.11
Wang et al., 2020 [38]	China	A, D	41	7.3	*E*	29.37
Wee et al., 2020 [34]	Singapore	A	445	2.2	*ORF1b-nsp14, E*	32.69
Wei et al., 2020 [44]	China	A	112	39.2	*Orf1ab, N*	-
Wu et al., 2020 [45]	China	A, C, F, I	200	19.0	*N, E, RdRp*	-
Ye et al., 2020 [18]	China	A	626	13.5	*Orf1ab, N*	-
Zhang et al., 2021 [12]	China	A; H	86	8.1	*E, RdRp*	-

Notes: Author – Author identification; Country – the county where the study was sampled; sampling site - location of the sampling study: A – COVID-19 Ward Hospital, B – Hemodialysis room, C – Virology Laboratory, D – Wastewater, E – River water; F – Intense Care Unit. Sample; I – Evaluation unit; J – Public environment; Sample Nº – Number of samples collected; % - Frequency of positive RT-qPCR samples considering the Samples Nº column; Target genes – genes target on the RT-qPCR; Low-Ct – Low Ct value found by these studies; [A] = Absence of difference among the chosen target gene and the frequency of positive results considering each study (p-value = 0.3732). References according to manuscript.

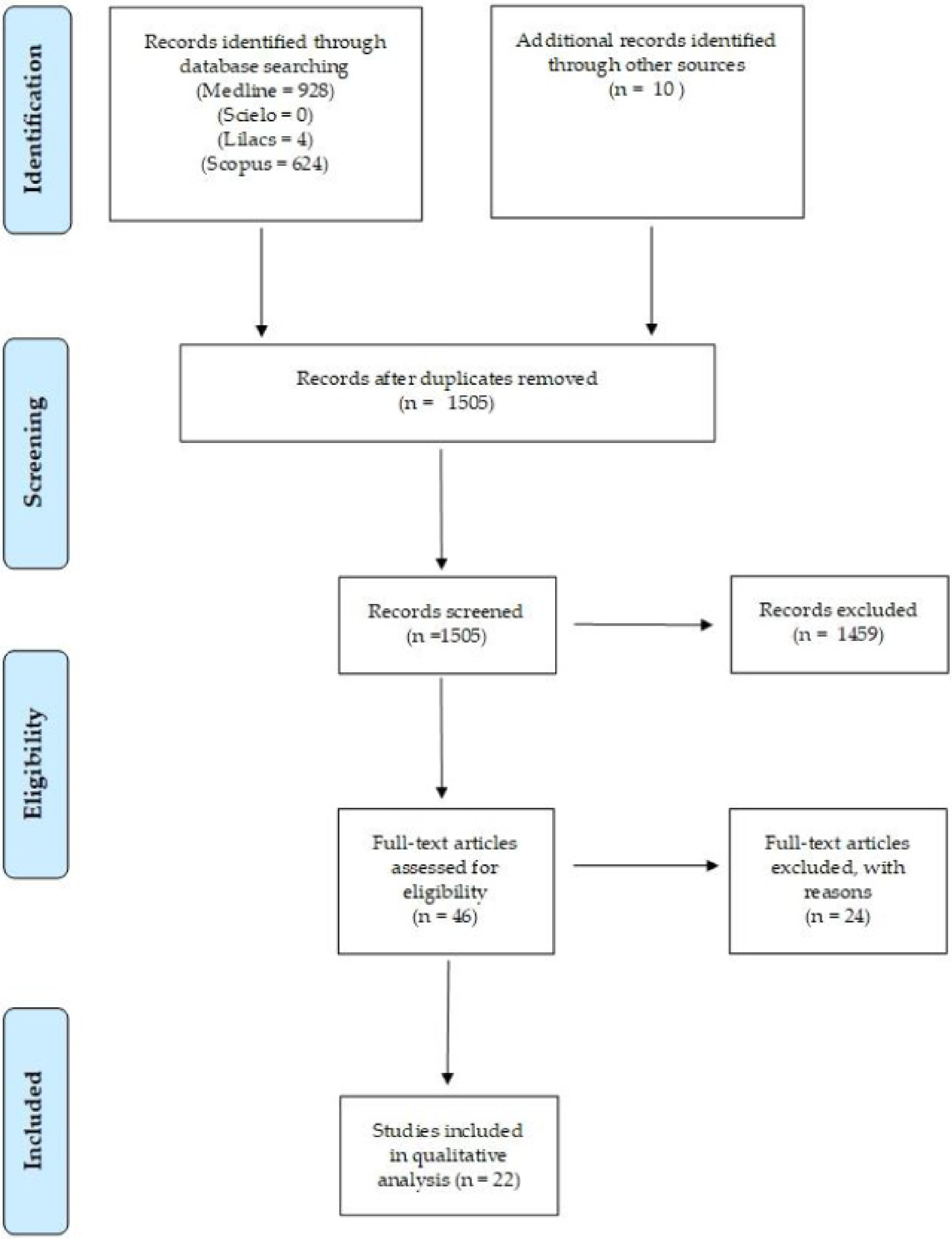

Supplementary Figure S1: Flowchart of search methods for bibliographic review. Methods of systematic Literature Review by PRISMA.

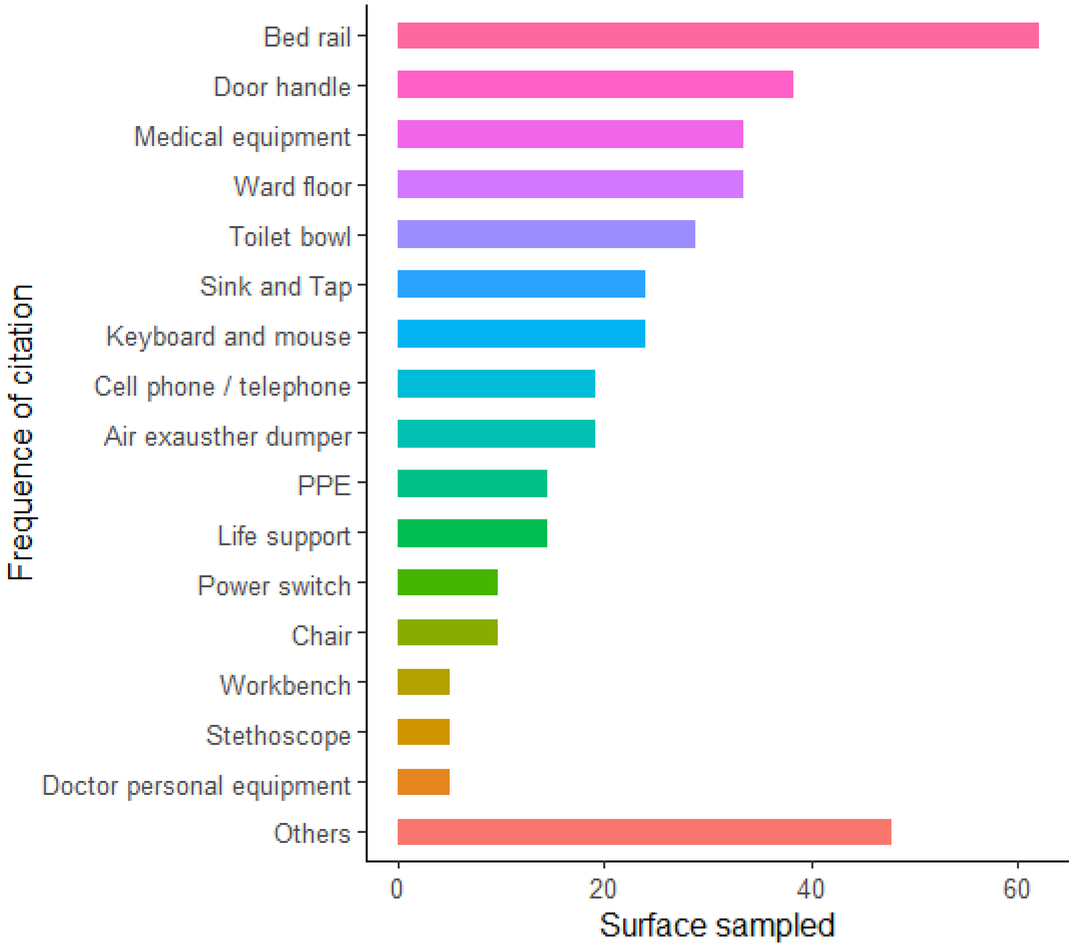

Supplementary Figure S2. Frequency of citation of the contaminated surface in the published data. As most papers did not specify the frequency of contamination of their sampled surfaces, the data collated show the frequency (number of citation/number of studies) of a surface being cited as contaminated by SARS-CoV-2 among the 22 studies included. Notes: Toilet = Foot flush button, toilet seat, toilet faucet, toilet bowl; Medical equipment = Cardic table, CPA helmets, Iodine bottle cap, surface of blood gas analyzer, ultrasonic machine button, canal of stomach, flashlight surface, catheter interface, channel surface of ECOM, touch screen, ambu bag, Infusion pump; PPE = Personal Protective Equipment; Others = Public places, windows glass, windows shield, trapeze bar, call button, upper part of the TV.
